# A Survey of Public Opinion on Cat (*Felis catus*) Predation and the Future Direction of Cat Management in New Zealand

**DOI:** 10.3390/ani7070049

**Published:** 2017-07-03

**Authors:** Jessica K. Walker, Stephanie J. Bruce, Arnja R. Dale

**Affiliations:** 1 New Zealand Companion Animal Council, P.O. Box 4, Waiuku, Auckland 2341, New Zealand; 2 Environmental and Animal Sciences Network, Unitec Institute of Technology, Auckland 1025, New Zealand; stephaniejean.bruce@gmail.com; 3 Royal New Zealand Society for the Prevention of Cruelty to Animals, 3047 Great North Road, New Lynn, Auckland 0610, New Zealand; arnja.dale@spca.nz

**Keywords:** companion cat, cat management, cat predation, feral cat, stray cat, National Cat Management Strategy, New Zealand, public opinion

## Abstract

**Simple Summary:**

The need to balance the benefits of cat ownership with the prevention of wildlife predation in New Zealand evokes strong and opposing views. This paper evaluates public concern for wildlife predation by four categories of cats; owned cats, managed-stray cats, unmanaged-stray cats, and feral cats. In addition, public support for a National Cat Management Strategy and a range of management techniques are investigated. Although the participants expressed concern regarding wildlife predation by all four categories of cats, the highest levels of concern were predation by feral cats, followed by unmanaged stray cats, then managed stray cats, and finally owned cats. The large majority of participants were found to support the implementation of a National Cat Management Strategy. Management techniques for owned cats that obtained public support included; cat exclusion zones, limits on ownership numbers, microchipping, Council registration, and de-sexing. Trap-Neuter-Return (TNR) was the favoured management technique for managed stray cats, while TNR and lethal management techniques were equally favoured for unmanaged stray cats. Lethal control methods were favoured for feral cats. The findings presented in this paper will be useful to consider during the development of legislation relating to cat management and predation in New Zealand.

**Abstract:**

Cat predation is a prominent issue in New Zealand that provokes strong and opposing views. We explored, via 1011 face-to-face questionnaires, public opinion on (a) support for a National Cat Management Strategy (78% support); (b) concern regarding predation of wildlife by owned and un-owned cats (managed stray, unmanaged stray, and feral cats); (c) the acceptability of management techniques for owned cats; and (d) the acceptability of population management techniques for un-owned cats. The highest concern was expressed regarding the predation of non-native and native wildlife by feral cats (60 and 86% repectively), followed by unmanaged stray cats (59 and 86% respectively), managed stray cats (54 and 82% respectively), and finally owned cats (38 and 69% repectively). Limits to the number of cats owned and cat restriction zones received high levels of support (>65%), and compulsory microchipping, Council registration, and de-sexing were supported by the majority (>58%). Public support of population control methods for unowned cats was explored, and the influence of participant demographic variables on responses is described. These findings provide insight into public opinion regarding the management of cats in New Zealand, which should be considered during the development of legislation in this area.

## 1. Introduction

Cats are widely kept and popular companion animals that offer significant benefits to their human owners while simultaneously having the potential to have a negative impact on society in general, as a result of a lack of legislation regarding their management. In New Zealand, 35–44% of households have at least one companion cat, making them the country’s most popular companion animal [1,2]. The presence of, and interaction with, companion cats is well documented to enhance the health and wellbeing of humans (e.g., [3,4,5,6]). Conversely, unregulated cat management is associated with negative societal effects, including the predation of wildlife and a reduction in the abundance of native species [2,7,8] of high cultural value, disease transmission [9], interbreeding and contribution to stray cat populations [10], nuisance behaviours (including fouling, fighting, and spraying) [11,12], and a risk of injury and death as a result of fighting, dog attacks, and traffic accidents [12,13]. In New Zealand, cats are legislatively categorised into three groups; companion cats, stray cats, and feral cats [14], and for the purposes of this study these are defined as follows:

1. Companion cats: domestic cats that live with humans and are dependent on humans for their welfare [14].
2. Stray cats: companion cats that are lost or abandoned and are living as an individual or in a group. Stray cats live around centres of human habitation and have their needs provided by for, either directly or indirectly, by humans.
3. Feral cats: not stray or owned and have none of their needs provided for by humans. Feral cats generally do not live around centres of human habitation [14].

Currently there are an estimated 1.134 million owned companion cats in New Zealand [1]. One study by Mahlow and Slater [15] has suggested that stray cat populations equate to 14% of the owned companion cat population, and this calculation has been used on a single occasion to estimate the stray cat population numbers in New Zealand to be 196,000 [16]. Feral cat population numbers have not been accurately quantified as feral cat densities vary widely across New Zealand.

Predation by cats is a prominent issue in New Zealand that evokes strong and opposing views. The number of prey items killed annually by New Zealand’s companion cat population has been approximated to be between 18 and 44 million, which has been calculated based on 35% of the companion cat population being active hunters [16]. Other studies suggest the rate is higher with between 44 and 70% of companion cats thought to be active hunters [2,8]. It is suggested that between 14 and 33 million prey are killed by stray cats annually [16]. Feral cat data is not reported as a result of the difficulties of accurately quantifying their population numbers. Although these estimated predation levels have not been validated in New Zealand, international predation levels have been demonstrated to affect the abundance of prey species [17,18]. Cats are opportunistic predators capable of killing a wide range of species [8]. While it is suggested that mammal species such as mice and rats account for the majority of cat prey items, avian, reptilian, amphibian, and invertebrate species can also be the targets of cat predation, making cats generalist predators as well as opportunists [19,20,21]. Their generalist nature means that cats are not limited by the abundance of one prey species, allowing them to drive populations and in some cases entire species, to extinction [20,22]. This is of particular importance in New Zealand, where many native fauna species are vulnerable to predation as a result of their evolving in environments without mammalian predators [22,23].

Cats’ abilities to predate and the negative impacts that this can have on New Zealand’s native fauna may be ameliorated by the implementation of a National Cat Management Strategy that includes regulatory management methods which aim to reduce the predation of wildlife. One such method is the employment of cat exclusion zones, areas that surround ecologically sensitive locations where residents are prohibited from owning cats [23,24] or where residents are required to keep cats indoors at all times. By reducing the number of cats in these areas, it is hoped that the negative impact they have on wildlife will also be reduced [24]. Research has documented that cats living near native populations hunt these species in larger quantities than cats that do not [12,24]. Restricting the number of cats allowed per household is another possible management technique, as fewer cats should theoretically equate to reduced predation impacts [24,25]. Cat curfews require owners to confine their cats indoors, whether it be during the day or night or continuously. In addition to reducing the risk of injury or death resulting from road accidents [13], confinement reduces the number of interactions between cats and prey species, potentially reducing predation levels [26]. Mandatory de-sexing, micro-chipping, and registration of cats are suggested key components of responsible companion cat ownership [27,28]. While these methods do not directly reduce predation levels, they do reduce the chance that companion cats will contribute to stray and feral populations through breeding, abandonment, or becoming lost [8,29,30]. Trap-Neuter-Return (TNR) programmes capture stray cats, de-sex and vaccinate them, and then return them to their capture site. Often these programmes will be carried out in conjunction with the adoption of socialised individuals [31,32]. Using cat exclusion zones, compulsory micro-chipping, and de-sexing in conjunction has been suggested to be the most effective way to manage cat populations in terms of reducing predation levels [8,25].

A National Cat Management Strategy may achieve a balance between the significant benefits of cat ownership with the aforementioned negative societal and environmental impacts of cats [26]. Understanding public concern regarding cat predation and attitudes towards management techniques is an important consideration when creating a management strategy [25]. Studies conducted internationally suggest that public opinion regarding cat predation and possible management techniques differ based upon a number of demographic variables including; knowledge and experience, employment status, beliefs and values and in some cases gender [12,25,33,34]. While little research in this area had been conducted in New Zealand, one notable exception is a recent study by Hall et al. [35], in which 347 New Zealand participants contributed to an online survey which aimed to elucidate attitudes towards companion cat predation across six countries. The results suggested that in New Zealand non-cat owners were more supportive of the need for cat legislation than cat owners.

Given the limited research investigating the palatability of national legislation surrounding the management of cats in New Zealand, we aimed, through the administration of a face-to-face questionnaire, to explore public opinion on:

1. A range of management measures thought to contribute to the reduction of risk to wildlife, and;
2. Demographic variables that influence these opinions.

## 2. Materials and Methods

### 2.1. Participants

Public opinion on cat predation and management was explored via 1016 face-to-face interviews in three central Auckland locations and two upper North Island rural towns. The surveying took place in central shopping areas. A team of 14 research assistants approached 8485 (12% response rate) members of the general public using simple random sampling [36] and asked them to participate in a 10-minute “social attitudinal survey”. Further details about the study were not provided to avoid the potential bias of attracting participants with a greater empathy or interest in cats. Before commencing the survey, the participants were provided with an information sheet outlining the length of the survey, the anonymity and confidentiality of the information they provided, and their right to withdraw from the survey at any time including up to four months after completion of the survey. After completing the survey participants were provided with a take home information sheet which included a unique number identifier that participants could use if they wished to withdraw their responses to the survey at a subsequent date. All aspects of the research were provided by the Unitec Research Ethics Committee, Auckland, New Zealand (2015–1083). A written script was followed during questioning to ensure the accurate and standardised delivery of the questions. Five questionnaires were discarded due to partial completion.

### 2.2. Questionnaire Design

A pilot study was conducted using five randomly selected participants in central Auckland. Based on this, some questions were altered for ease of understanding. The final questionnaire consisted of 62 questions that were separated into three sections, namely, (a) cat predation and management, (b) cat ownership; and (c) participant demographics, and were asked in that order to reduce bias from questions about cat ownership influencing answers to subsequent questions about cat management. Cats were categorised into one of four sub-groups to allow information pertaining to methods of population management to be differentiated for each.

“A companion cat (1) is a common domestic cat that lives with humans and is dependent on humans for its welfare” [14].

“A stray cat is a companion cat that is lost or abandoned and that is living as an individual or in a group. Stray cats live around centres of human habitation. There are two categories of stray cat, colony and unmanaged. Colony cats (2) have many of their needs, such as food and shelter, directly supplied by humans. Unmanaged stray cats (3) have their needs supplied indirectly by humans by scavenging etc.”

“A feral cat (4) is a cat that is not stray or owned and that has none of its needs provided for by humans. Feral cats generally do not live around centres of human habitation” [14].

The questions in section (a) related to the perceived impact of predation by each different cat sub-group and the use of different techniques to manage New Zealand’s populations of these cat sub-groups in turn. Firstly, the participants were asked if they thought New Zealand should have a National Strategy in place to manage cat populations. No information was provided regarding what a National Cat Management Strategy might include. Participants were then asked whether they were concerned about the predation of both native and non-native wildlife by each of the four sub-groups of cats. For the companion cat sub-group, participants were asked an additional 11 questions relating to their management. These included whether there should be a limit to the number of cats one household can own at one time and what the limit should be; whether de-sexing, microchipping, and registration should each be compulsory; whether cats should be confined to their owners’ property or inside their owners home and at what times (options: always, day, night, other); and whether there should be certain areas that companion cats should not be allowed to be owned and where these areas should be. For the colony, unmanaged stray, and feral cat subgroups, three additional questions were asked about their management. These included whether action should be taken to control each subgroups population numbers and, if so, who should be responsible for controlling these populations (options: Government, Council, Society for the Prevention of Cruelty to Animals (SPCA), other).

The participants were asked how each group of cats should be controlled. This question was open-ended and the responses were categorised following data collection into one of the following five categories: (1) lethal methods (e.g., shooting); (2) Trap-Neuter-Return (TNR); (3) “non-killing” methods (e.g., de-sexing, socialising, and rehoming); (4) other (do nothing, leave cats alone); and (5) don’t know.

After the respondents described their chosen method of population control, they were read a definition developed by the research team for lethal methods of population control and TNR. Following this, they were given the opportunity to change their chosen population control method to one of three posited options (“lethal methods”, “TNR”, or “nothing”). The definitions were as follows:

“Lethal methods can be employed to kill cats and reduce population numbers. These methods may include poisoning and kill trapping”.

“Trap-Neuter-Return is a program through which stray and feral cats are trapped humanely; de-sexed and medically treated; and then returned to the location where they were found”.

“Nothing—leave the cat populations as they are”.

The categories, “other” and “nothing” were combined into one category, “other”, for comparative data analysis due to the small number of respondents selecting “nothing” and because “leave the cats alone” and “do nothing” were common responses observed in the “other” category.

The questions in section (b) covered demographic information about the number of participants that owned companion cats (32%; *n* = 326), including the total number of cats each participant owned (median = 1), the length of ownership (the median was seven years), the reasons for ownership (companionship 54%; pest control 4%; other 42%), how the cats were housed (indoor 13%; outdoor 7%; both 80%), the number that wore collars (35%), the number that were de-sexed (94%), the number that were microchipped (37%), and the number of microchips that were registered (81%). The participants were also asked if they owned other companion animals (yes = 26%) or had owned companion animals during the previous five years (yes = 39%) or during childhood (yes = 85%). The respondents who did not own a cat were asked to elucidate as to why. This question was open-ended and responses were categorised following (living situation not suitable 26%; allergies 4%; lifestyle 19%; don’t want/like 11%; have other animals 6%; last one passed away 6%; other 28%).

The third and final section of the questionnaire collected demographic information about each participant. All age categories were well represented, with a slightly higher representation of 18–35 year olds. Male and female participants were equally represented. The majority of participants were New Zealand European and 45% were single, with an equal number either married or in de facto relationships. Over 70% of participants lived in urban/suburban locations with 14% living rurally. Most earned over $50,000 per annum and 70% were employed. The majority were well educated, with over 75% having attained tertiary qualifications. Taken together, these demographic data indicate that our sample population showed similar trends to the most recent New Zealand census data (see Table 1).

The questions used in this research were based upon those of similar studies (e.g., [25,38]) and were written in a neutral manner so as not to influence participants’ answers with negative or positive wording [34]. Unless otherwise stated, respondents were asked to answer “yes”, “no”, or “don’t know” to all posited questions. Three versions of the questionnaire were developed so that the cat categories in section (a) were rotated between participants to prevent delivery fatigue. In an effort to reduce participant confusion, the definition of each of the cat sub-groups was repeated before the questions were asked.

### 2.3. Statistical Analysis

All questionnaire data was entered into Microsoft Excel and then exported into Minitab Statistical Software (version 17, Minitab Pty Ltd, Sydney, Australia). Cross-tabulation with a Chi-Squared (χ^2^) test of association was used to investigate the differences in participant concern regarding the predation by each of the cat groups.

Nominal logistic regression was used to investigate associations between demographic variables and each question about the management of cats in New Zealand. Unanswered questions were coded as missing data. The regression model was refined using a backwards stepwise technique, sequentially removing non-significant predictors and refitting to identify which predictors were important. The final model was as follows:

*Z* = b_0_ + b_1_X_1_ + b_2_X_2_ + b_3_X_3_ + b_4_X_4_ + b_5_X_5_ + b_6_X_6_ + b_7_X_7_ + b_8_X_8_

where *Z* is the log odds of the dependent variable; b_0_ is a constant; b_1_ is the coefficient for companion cat ownership (X_1_); b_2_ is the coefficient for age (X_2_); b_3_ is the coefficient for gender (X_3_); b_4_ is the coefficient for ethnicity (X_4_); b_5_ is the coefficient for marital status (X_5_); b_6_ is the coefficient for residential location (X_6_); b_7_ is the coefficient for income (X_7_); and b_8_ is the coefficient for education (X_8_).

The above regression model was used for each of the questions of interest in the survey. For all models, validity was assumed with a log-likelihood *p* value of <0.05 and Goodness of Fit Pearson and Deviance chi-squared *p* values of >0.05. The results are presented as odds ratios (OR) and *p* values. Only significant results are presented.

## 3. Results

### 3.1. A National Cat Management Strategy

When asked whether New Zealand should have a National Cat Management Strategy, 78% of participants responded “yes” (“no” = 12%; “don’t know” = 9%). The participant responses were found to be influenced by several demographic variables, including annual income, ethnicity, and residential location. Participants earning in excess of $100,000 per annum were more likely to respond “no” (Z = 2.51, OR = 2.84, *p* = 0.012, CI = 1.26–6.39) than participants earning less than $50,000 per annum. Participants of Pacific/Cook Island ethnicity were more likely than NZ Europeans to respond “don’t know” (Z = 2.03, OR = 4.09, *p* = 0.042, CI = 1.05–15.92), and participants residing in urban locations were less likely to respond ‘yes’ than suburban respondents (Z = −1.49, OR = 0.49, *p* = 0.028, CI = 0.25–0.93).

### 3.2. Cat Predation

**Figure 1:**
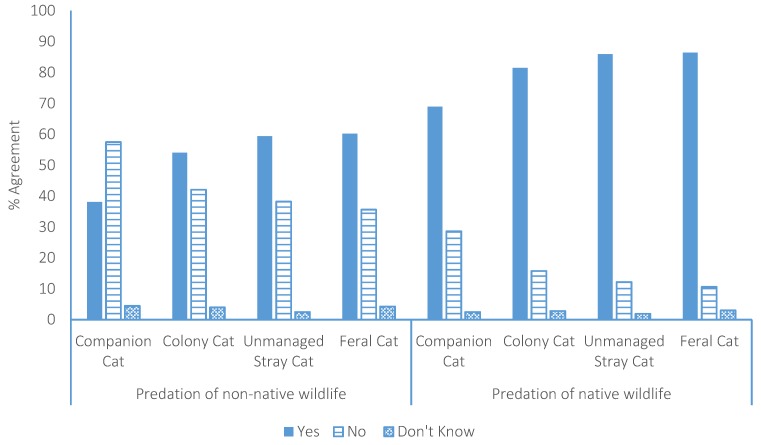
Participant concern about the predation of non-native and native wildlife by each sub group of cat in New Zealand.

The participants were asked whether they were concerned about the predation of non-native and native wildlife by each of the four categories of cat. The participants expressed greater concern about the predation of native wildlife than non-native wildlife by all four cat groups (χ^2^ = 792.4, df = 1, *p* < 0.0001). In the case of non-native wildlife, the participants were more concerned about predation by colony cats (54%; χ^2^ = 51.6, df = 1, *p* < 0.0001), unmanaged stray cats (59%; χ^2^ = 91.5, df = 1, *p* < 0.0001), and feral cats (60%; χ^2^ = 98, df = 1, *p* < 0.0001) than predation by companion cats (38%) and more concerned about predation by unmanaged stray cats (χ^2^ = 5.9, df = 1, *p* = 0.015) and feral cats (χ^2^ = 7.8, df = 1, *p* = 0.005) than by colony cats (Figure 1). With regard to native wildlife, participants were more concerned about the predation of native wildlife by colony cats (82%; χ^2^ = 42.8, df = 1, *p* < 0.0001), unmanaged stray cats (86%; χ^2^ = 83.8, df = 1, *p* < 0.0001), and feral cats (86%; χ^2^ = 89.4 df = 1, *p* < 0.0001) than predation by companion cats (69%) and more concerned about native wildlife predation by unmanaged stray cats (χ^2^ = 7.4, df = 1, *p* = 0.007) and feral cats (χ^2^ = 9.2, df = 1, *p* = 0.002) than by colony cats (Figure 1).

Concern regarding the predation of both non-native and native wildlife by each of the different categories of cat was found to be influenced by age, gender, ethnicity, income, residential location, marital status, and education (Supplementary Materials Table S1). Younger respondents were less likely to be concerned about predation by companion cats (*p* < 0.0001; *p* < 0.0001, respectively); colony cats (*p* < 0.0001; *p* = 0.004, respectively); unmanaged stray cats (*p* = 0.001; *p* = 0.001, respectively); and predation solely of non-native wildlife by feral cats (*p* = 0.002). Males are more likely to express concern about the predation of native wildlife by companion cats than are females (*p* = 0.038). Participants of NZ European ethnicity were less likely than those of Asian/Indian ethnicity to be concerned about the predation of non-native wildlife by companion cats (*p* < 0.0001), colony cats *p* = 0.001), unmanaged stray cats (*p* = 0.001), and feral cats (*p* = 0.010) and more likely to be concerned about the predation of native wildlife by colony cats (*p* = 0.030) than participants identifying as “other” ethnicities. Participants earning greater than $100,000 per annum were more likely than those earning less than $50,000 per annum to be concerned about the predation of native wildlife by unmanaged stray cats (*p* = 0.016), while those who elected not to disclose their annual income were more likely than those earning less than $50,000 per annum to be unsure whether they were concerned about non-native (*p* = 0.02) and native (*p* = 0.014) predation by colony cats. Rural participants were more likely to be concerned about the predation of non-native wildlife by unmanaged stray cats (*p* = 0.047) than were urban participants. Married participants were more likely than single participants to be concerned about the predation of non-native wildlife by colony cats (*p* = 0.004) and unmanaged stray cats (*p* = 0.05), and participants with primary or no formal education were less likely than participants with a certificate/diploma (*p* = 0.039), undergraduate (*p* = 0.021), or postgraduate (*p* = 0.017) qualification to be concerned about the predation of native wildlife by colony cats. These participants were also more likely than participants with secondary education (*p* = 0.046) and postgraduate education (*p* = 0.038) to respond “don’t know” when asked if they were concerned about the predation of non-native wildlife by feral cats.

### 3.3. Management of Companion Cats

The participants were asked three questions regarding the responsible management and identification of companion cats. Fifty-eight percent (*n* = 584) of participants responded “yes”, it should be compulsory for companion cats to be de-sexed, while 66% (*n* = 667) responded “yes”, it should be compulsory for companion cats to be microchipped, and 61% (*n* = 616) responded “yes”, it should be compulsory for companion cats to be registered with the Council.

Demographic variables (Supplementary Materials Table S2) were found to influence the responses. Support for compulsory de-sexing was influenced by gender, with male participants being less supportive than females (*p* = 0.031). Age influenced agreement with compulsory de-sexing, with increasing support observed as age reduced (*p* < 0.0001), and compulsory microchipping, with increasing age corresponding to increased uncertainty (*p* = 0.019). Support for compulsory microchipping was also influenced by residential location, with suburban residents more likely to be uncertain (*p* = 0.018), and marital status, with married participants demonstrating less support than single participants (*p* = 0.024). Mandatory Council registration was influenced by cat ownership, with owners demonstrating less support (*p* < 0.0001); ethnicity, with NZ Europeans being less supportive than participants of Asian/Indian ethnicity (*p* = 0.002); and age, with older participants demonstrating more support (*p* < 0.0001).

**Figure 2:**
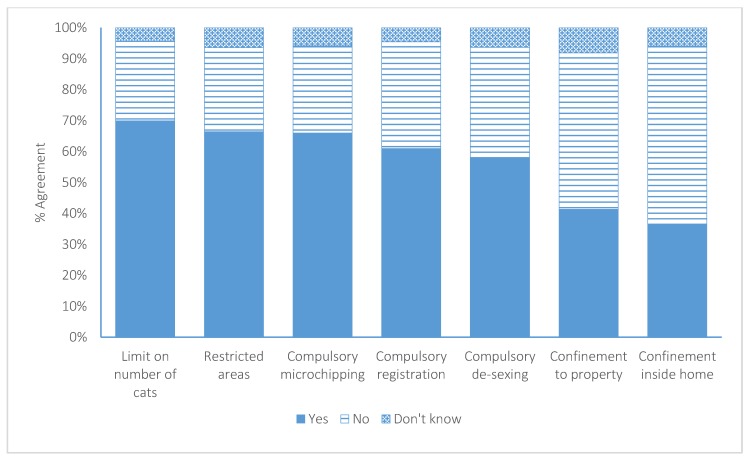
Participant agreement to seven posited restrictions regarding companion cat ownership in New Zealand.

The participants were asked a further four questions relating to restrictions on cat ownership (Figure 2). Seventy percent (*n* = 706) agreed that there should be a limit to the number of cats per household.

Forty-one percent (*n* = 417) agreed there should be times when cats must be confined to their owner’s property, of which 8% believed this should occur during the day, 45% at night, 19% at all times, 25% at other times, and 3% did not respond. Thirty-six percent (*n* = 368) agreed there should be times when cats must be confined inside their owner’s homes, of which 27% agreed this should occur at all times, 6% during the day, 54% at night, 11% at other times, and 1% did not respond. The majority of participants (66%; *n* = 671) agreed there should be areas that companion cats are not allowed to be owned, of which 70% suggested this should be in areas near endangered/protected species, 5% near human habitation (e.g., apartments, urban areas), 15% other, and 10% did not answer.

The responses to these questions were influenced by a number of demographic variables (Supplementary Materials Table S2). Support for a restriction on the number of cats each household could own was influenced by age, with decreased support observed as age reduced (*p* = 0.047); marital status, with single participants less supportive than married participants (*p* = 0.009); education, with participants that had only primary or no formal education demonstrating less support than those with a certificate or diploma (*p* = 0.030); and ethnicity, with NZ Europeans being more supportive (*p* = 0.026) than Europeans. Support for the confinement of cats to their owner’s property was influenced by ownership, with owners being less supportive (*p* = 0.008), and ethnicity, with NZ Europeans less supportive than Māori (*p* = 0.039), Asian/Indian (*p* < 0.001), and Pacific/Cook Islanders (*p* = 0.028). On the other hand, the confinement of cats inside their owners’ homes was influenced by age, with decreased support observed as age reduced (*p* < 0.0001); income, with those earning more than $100,000 per annum demonstrating more support (*p* = 0.019); and ethnicity, with NZ Europeans being less supportive than Asian/Indian (*p* < 0.0001) and Pacific/Cook Islanders (*p* = 0.004). Support for areas of exclusion of companion cat ownership was influenced by cat ownership, with cat owners demonstrating less support (*p* < 0.0001); ethnicity, with NZ Europeans being more supportive than Asian/Indian (*p* = 0.021) and Europeans (*p* = 1.71); and income, with those earning between $50,000–$100,000 per annum (*p* = 0.008) or greater than $100,000 per annum (*p* = 0.033) being less likely to be unsure than participants earning less than $50,000 per annum.

### 3.4. Management of Colony Cats

**Figure 3:**
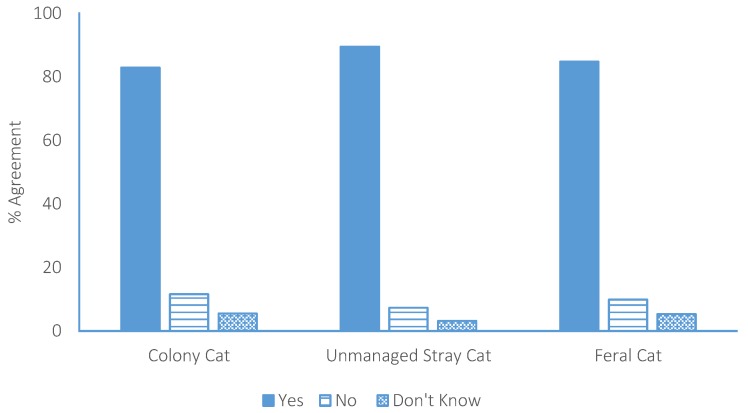
Participant agreement that action should be taken toward controlling each of the un-owned subgroups of cats in New Zealand.

**Figure 4:**
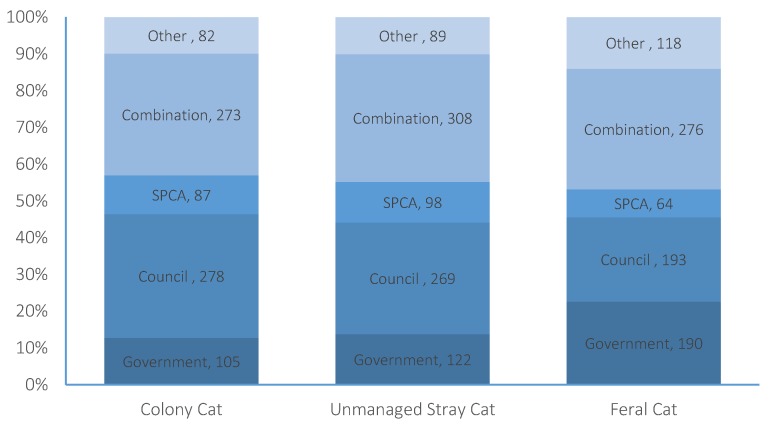
Participant opinion as to which organisation should be responsible for the control of each subgroup of un-owned cats in New Zealand.

**Figure 5:**
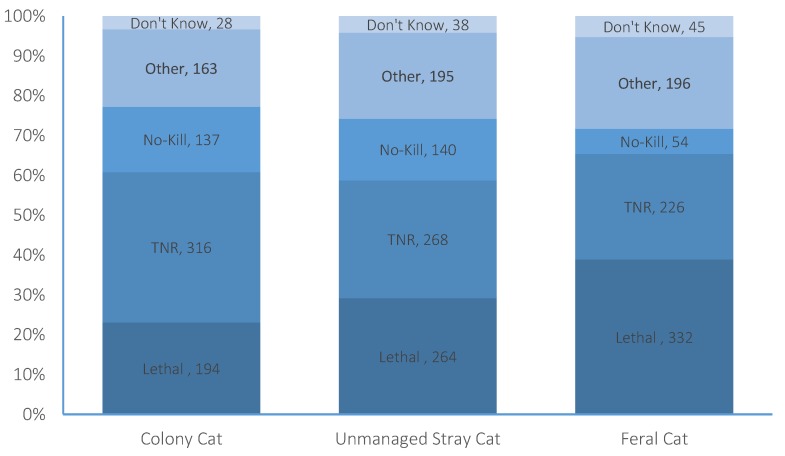
Participant opinion on the action that should be taken to control each subgroup of un-owned cats in New Zealand.

The participants were asked three questions regarding the management of each of the three categories of un-owned cats. In the case of colony cats, 83% (*n* = 825) of the participants agreed that action should be taken towards controlling colony cat populations (Figure 3). Of these, 13% believed the Government should be responsible for controlling these populations, 34% local Councils, 10% SPCA, 33% a combination of all three, and 10% selected “other” (Figure 4). When asked what action should be taken towards controlling colony cats, 20% agreed that lethal methods should be engaged, 7% selected TNR, 28% selected non-killing methods (e.g., rehoming), 32% selected “other”, and 13% selected “don’t know”. Following the explanation of lethal methods and TNR, 33% (*n* = 336) indicated that they would like to change their answer, resulting in 23% agreement with lethal methods, 38% agreement with TNR, 16% agreement with “non-killing” methods, 20% agreement with “other” methods, and 3% selecting “don’t know” (Figure 5).

Responses regarding responsibility for colony cat control were found to be influenced by a number of demographic variables (Supplementary Materials Table S3), including ownership, with non-owners being more likely to agree that the SPCA should be the organisation responsible (*p* = 0.048); ethnicity, with NZ Europeans less likely than Asian/Indian (*p* = 0.022), European (*p* = 0.033), and “other” ethnicities (*p* = 0.02) to agree that the Government should be responsible; residential location, with suburban participants being more likely than urban participants to agree that a combination (i.e., SPCA, local Council, and Government) (*p* = 0.012), the SPCA (*p* = 0.012), or the Council (*p* = 0.04) should be responsible; and income, with those earning less than $50,000 per annum being less likely than those with an income bracket of $50,000–$100,000 or greater than $100,000 to agree that the Government (*p* = 0.03; *p* = 0.022, respectively) should be responsible. Demographic variables also influenced participant opinion on the type of control method that should be utilised for colony cats (Supplementary Materials Table S3), including ownership, with owners being less supportive of lethal methods than TNR (*p* = 0.032) or “non-killing” methods (*p* = 0.025); age, with younger participants being less supportive of lethal methods (*p* < 0.0001); gender, with females demonstrating more support for non-killing methods over lethal methods (*p* = 0.026); residential location, with urban residents being less supportive of lethal methods than were rural residents (*p* = 0.001); and income, with those earning less than $50,000 per annum being less supportive of lethal methods than those with an income bracket of $50,000–$100,000 (*p* = 0.006) or greater than $100,000 (*p* = 0.006).

### 3.5. Management of Unmanaged Stray Cats

When participants were asked the same three questions regarding the management of unmanaged stray cats, 90% (*n* = 905) of participants agreed that action should be taken towards controlling populations (Figure 2). Of these, 14% believed that the Government should be responsible for their management, 30% local Councils, 11% the SPCA, 35% a combination of all three, and 10% selected “other” (Figure 3). When asked what action should be taken towards controlling unmanaged stray cats, 27% agreed that lethal methods should be engaged, 17% selected TNR, 19% selected non-killing methods, 25% selected “other”, and 12% selected “don’t know”. Following the explanation of lethal methods and TNR, 16% (*n* = 163) indicated that they would like to change their answer, resulting in 26% agreement with lethal methods, 26% agreement with TNR, 14% agreement with “non-killing” methods, 19% agreement with “other” methods, and 4% (*n* = 38) of participants selecting “don’t know” (Figure 4).

Demographic variables were found to influence responses (Supplementary Materials Table S3), including age, with younger participants being less likely to agree that the control of unmanaged stray cat populations should occur (*p* = 0.014); ethnicity, with NZ Europeans being less likely than participants identifying with ”other” ethnicities to agree that control should occur (*p* = 0.004); and income, with participants earning less than $50,000 per annum, compared to those earning greater than $100,000 per annum, being more likely to agree control should occur (*p* = 0.001). The organisation believed by participants to be responsible for the control of unmanaged stray cat populations was influenced by gender, with males being less likely to agree that control should be provided by a combination of organisations (*p* = 0.025); ethnicity, with NZ Europeans being less likely than Asian/Indian participants to agree that the Government should provide control (*p* = 0.008); income, with those earning more than $100,000 per annum being less likely to agree that the SPCA should provide control (*p* = 0.007); and residential location, with suburban residents being more likely than urban residents to agree that a combination of organisations should provide control (*p* = 0.022). The selected method of control was influenced by age, with younger participants being less likely to select lethal methods over TNR (*p* < 0.0001), “non-killing” methods (*p* < 0.0001), “other” methods (*p* < 0.0001), or “don’t know” (*p* = 0.001); ethnicity, with Asian/Indian respondents being more likely than NZ Europeans to select “non-killing” methods (*p* = 0.049), and “other” methods (*p* = 0.017) over lethal methods and Europeans being more likely to select “non-killing” methods over lethal methods (*p* = 0.045) than were NZ Europeans; income, with those earning between $50,000 and $100,000 per annum (*p* = 0.003) and those earning greater than $100,000 per annum (*p* = 0.002) being less likely than those earning less than $50,000 to select lethal methods over TNR; marital status, with single participants being less likely than those in de facto relationships to select lethal methods over TNR (*p* = 0.007) or “non-killing” methods (*p* = 0.002); and residential location, with urban participants being less likely than rural participants to select lethal methods over “other” methods (*p* = 0.05) of control.

### 3.6. Management of Feral Cats

Eighty-five percent of participants (*n* = 857) agreed that action should be taken towards controlling feral cats. Of these, 22% believed that the Government should be responsible for their management, 23% local Councils, 8% the SPCA, 33% a combination of all three, and 14% selected “other”. When asked what action should be taken towards controlling feral cats, 39% agreed that lethal methods should be engaged, 9% selected TNR, 8% selected non-killing methods, 17% selected “other”, and 11% selected “don’t know”. Following the explanation of lethal methods and TNR, 22% (*n* = 222) of participants indicated that they would like to change their answer, resulting in 33% agreement with lethal methods, 22% agreement with TNR, 5% agreement with “non-killing” methods, 19% agreement with “other” methods, and 4% selecting “don’t know” (Figure 4).

The responses to these questions were found to be influenced by a number of demographic variables (Supplementary Materials Table S3), including; age, with younger participants being less likely to agree that action should be taken towards controlling feral cats (*p* < 0.0001); and ethnicity, with Asian/Indian ethnicity (*p* = 0.044) and “other” ethnicities (*p* = 0.011) being more in agreement that action should be taken than were NZ Europeans. In addition, NZ Europeans were less likely than Asian/Indian participants to agree that the Government, rather than “other” organisations, should be responsible for controlling feral cats (*p* = 0.033). Ethnicity also influenced participants’ preferred method of control for feral cats, with Europeans (*p* = 0.001), Asian/Indians (*p* = 0.020), and Pacific/Cook Islanders (*p* = 0.015) all being more likely than NZ Europeans to select “non-killing” methods over lethal methods, while Māori participants (*p* = 0.020), Asian/Indian participants (*p* = 0.006) and Pacific/Cook Island participants (*p* = 0.029) were more likely than NZ Europeans to select “other” methods of control over lethal methods. Marital status also influenced control methods, with single participants being less likely than those that were married to select lethal methods over non-killing methods (*p* = 0.003) and less likely than participants in de facto relationships to select lethal methods over TNR (*p* = 0.014).

## 4. Discussion

The main aim of this study was to explore public support for a National Cat Management Strategy and opinions on posited cat management techniques. Our survey demonstrated strong public agreement (78%) that New Zealand should have a National Strategy in place to manage cat populations. Support for cat legislation has been similarly favoured in a number of Australian public opinion surveys [11,25,35,39], yet public support in New Zealand has recently been documented at only 55% [35]. This discrepancy might be explained by the small sample size (*n* = 347) and the middle class demographic of the participants involved in the recent study by Hall et al. [35].

### 4.1. Cat Predation

Our participants were concerned about the predation of both non-native and native wildlife, with the greatest level of concern expressed towards native wildlife. Concern regarding the predation of both non-native and native wildlife was highest for feral cats, followed by unmanged stray cats, then colony cats, and finally companion cats. Lower levels of concern regarding companion cat predation have been reported in previous studies (e.g., [25]) and appear to reflect a common misconception that companion cats have less or no impact upon wildlife populations [24]. This idea has been evidenced to be largely misguided, with well fed companion cats being just as likely to predate as other cat types [24,40].

The importance of preserving New Zealand’s native fauna species is evident on both a national and individual level and may explain the high levels of concern expressed regarding cat predation. In New Zealand many native fauna species are vulnerable to predation as a result of their evolving in environments without mammalian predators [20,23]. Native fauna preservation efforts are important for the culture and identity of many New Zealanders, with species such as the kiwi (*Apteryx* spp.) considered to be *tāonga* (treasure; something of value) to Māori and of significant national importance to all cultures within New Zealand [41]. Furthermore, the abundance of native fauna species is linked to the New Zealand economy through tourism, with approximately 90% of international visitors to New Zealand experiencing the natural surroundings [42,43]. Degradation of the country′s natural environments and a loss of iconic fauna species would result in an estimated loss of 4.9% GDP per annum received from the tourism sector [44].

Sixty-six percent of our participants supported the idea of cat ownership exclusion zones, a higher figure than has been reported in international studies [25,26]. New Zealand Europeans were more supportive of cat exclusion zones than participants of other ethnicities and demonstrated less concern regarding the predation of non-native wildlife. These results reflect the cultural importance of endemic species preservation within New Zealand.

We also found that male participants were more likely to demonstrate concern regarding the predation of native wildlife by companion cats. Gender is a well-established variable of influence when it comes to attitudes towards animals [45], with females frequently being reported to show more positive attitudes toward individual animals [46,47,48,49,50], while, on the other hand, males are reported to maintain a more utilitarian view of animals, expressing greater concern for species preservation and habitat conservation [45,51,52,53].

### 4.2. Management of Companion Cats

We observed only 58% public support for mandatory de-sexing. This was surprising as voluntary de-sexing levels for companion cats were reported at 94% in the present study and greater than 93% in previous New Zealand studies [1,35], 88–93% in Australia [11,25], and 86% in Singapore [54]. High levels (>70%) of support for mandatory de-sexing have also been observed for both owners and non-owners of companion cats in public opinion research internationally [25]. We found that males demonstrated less support for mandatory de-sexing, which is not surprising as females are frequently reported to be more in favour of de-sexing as a mechanism to control cat populations [25,39,54]. However, our almost equal proportions of male and female participants might go some way to explaining our seemingly low level of public support for mandatory de-sexing overall, as research investigating similar trends often describes female participant rates of 60–80% [25,54], which is likely to equate to higher levels of support for mandatory de-sexing.

Public agreement that microchipping should be compulsory in the present study was more than double (66%) the current 31% of companion cats microchipped within New Zealand [1] and much higher than the 37% of cats in the current study that were reported as microchipped. Unlike other methods of identification (e.g., collars and tags), microchipping is the only permanent and unalterable form of identification currently available for cats and gives cats a greater degree of protection and a much higher chance of being returned to their home when lost. Research has indicated that return-to-owner rates for cats that are microchipped is 20 times higher than for cats that are not microchipped [27]. Our results suggest that although participants were in favour of compulsory microchipping this was not being translated into practice. We found that older participants were more inclined to be unsure about compulsory microchipping which may be a reflection of a lack of understanding around the technology associated with microchipping. Further investigation is required to elucidate the discrepancy between public support for compulsory microchipping and the practice of microchipping.

Over 60% of respondents agreed that cats should be registered with local Councils. However this figure is lower than international levels of support for Council registration, which have been reported to be as high as 80% [25]. We found that non-owners were more likely to support the practice of registering cats with Councils. Likewise, ownership has been found to influence agreement regarding the control of cats in Australia, with non-owners demonstrating higher levels of support for mandatory registration with the Councils [25]. If mandatory Council registration of cats was to be included in a National Strategy within New Zealand, a demonstration of the benefits to cat owners might encourage compliance.

We asked participants whether there should be a limit to the number of cats per household and what that limit should be. There was 70% agreement with a limit on ownership numbers, with a median number of a two cat limit observed. This finding supports current cat ownership behaviour in New Zealand, where the average number of cats per household is 1.5 [1,35], and the median in the current study was one cat per household.

We observed low levels of public agreement regarding cat confinement. Less than half of the participants agreed that cats should be confined to their owner’s property, and only 36% agreed that cats should be confined inside the owner’s home. These findings are consistent with other studies in which constant indoor confinement has been found to be less widely accepted [25,35]. In one New Zealand study, the percentage of owned cats constantly confined was reported to be as low as 3% [55]. Of the participants that did agree with cat confinement, both to the owner’s property and inside the owner’s home, 45% and 54%, respectively, believed this should occur during the night. This finding parallels a recent study by Harrod et al. [56], who found that 48% of respondents agreed with the confinement of cats indoors overnight. Keeping companion cats continuously confined, as they are in some countries (50–60% in North America), should reduce their ability to hunt in comparison to that of unowned cats [57], yet keeping cats confined only at night may contribute little to reducing predation behaviour as research suggests that companion cats largely hunt during the day [24]. Unlike in other countries that are home to native nocturnal mammals vulnerable to predation by cats (e.g., Australia), introducing mandatory confinement overnight could result in a reduction of the number of rodent predations, which pose a significant threat to New Zealand native wildlife. Furthermore, compliance levels relating to the confinement of cats at night have been reported to vary between 32–80% in Australia [12,13]. Conversely, keeping cats confined at night would be beneficial for cat welfare as cat curfews have been documented to reduce the injury rates associated with road accidents, encounters with other cats, and dog attacks [12,13]. Our results suggest that age and ownership status are demographic variables that impact upon support for cat confinement, with older participants and non-owners being more likely to support mandatory confinement to the owner’s property. Both national [36,55] and international [12,25,39] research document non-owner support of the confinement of cats to their owner’s property, with the underlying motivation suggested to be based on the belief that containment is important to protect neighbours from nuisance behaviour [12]. If cat confinement or curfews are introduced in New Zealand as part of a National Cat Management Strategy, the benefits for both owners (i.e., reduced injury rates) and non-owners (i.e., reduced nusiance behaviour) would need to be illuminated in order to promote support and compliance.

### 4.3. Management of Unowned Cats

We found high levels of public agreement that action should be taken to control colony (83%), unmanaged stray (90%), and feral (85%) cat populations within New Zealand. A National Cat Management Strategy would likely need to be governed and enforced, and therefore participant opinion regarding which organisation(s) should be responsible for each subgroup of cats is an important part of the determination process. We found that participants were most likely to select the Council as the organisation that should be responsible for controlling colony cats, while all three organisations combined (Government, Council, and the SPCA) were favoured for controlling both unmanaged stray cats and feral cats. These results indicate that participants viewed unmanaged stray and feral cat population control on more of a nationwide basis, hence the multi-organisation seletion, but colony cat populations on an area by area basis, hence the Council control. New Zealand Europeans were less likely to believe that the Government should be responsible for colony cats and more likely to agree that lethal methods should be used to control feral cat populations, which might reflect the Department of Conservation’s (a Government department) historical and ongoing role in feral cat control within New Zealand.

After participants were read our definitions of lethal methods and TNR, they were asked if they would like to replace their free-answer to the question of how each group of unowned cats should be controlled. Overall we observed low levels of agreement amongst participants. TNR was the favoured population control method for colony cats, obtaining 31% support, while TNR and lethal methods were equally favoured for unmanaged stray cats at 26%, and lethal methods were favoured for feral cats at 33%. As much less than half of the study population was in agreement regarding control methods. Further research should be carried out to investigate public support for alternative methods. In New Zealand, feral cats are classified as pests; consequently lethal methods of control such as kill trapping and poisoning are employed to manage population numbers [33], which may explain the higher level of support for the use of lethal methods with feral cats. Similarly, it has been suggested that lethal methods may be used to control stray cat populations unless evidence of their “ownership” can be provided [33]. This may in part explain the division of favour between TNR and lethal methods in the case of unmanaged stray cats. We found that females were more likely to select TNR over lethal methods of control for all sub-groups of unowned cats. Similar findings have been found in previous studies where females have been reported to be less supportive of lethal control methods for both stray and feral cats [58] and more supportive of TNR as a management technique [59,60,61]. In order to gain the greatest level of support for the management techniques posited in a National Strategy, the techniques must be demonstrated to not only positively impact wildlife through reduced predation but do so in a manner that is considerate of the welfare of individual cats and their humane treatment. Similar to the demographic variables of influence reported in other research [58], we found that older participants and those living in rural locations were more accepting of lethal control methods. This could be a reflection of increased interaction with wildlife in rural areas or increased exposure to unowned cats in rural areas.

Furthermore, it is important to point out that based on our definition, TNR may have been interpreted by participants as a robust mechanism of population control for all categories of cats specified in our study that does not involve euthanasia. Consequently, participants may have favoured TNR when it was considered alongside lethal methods, which suggests a possible bias in our results. Although TNR programmes aim to create a stable population in which cats can no longer reproduce and natural attrition eventually results in decreased numbers [62], in reality, the effectiveness of TNR at reducing the population numbers of cats within a colony depends on a number of variables. These include the prevention of immigration of cats from outside of the colony, maintaining ongoing high levels of de-sexing of the individuals within the colony and any new immigrants, the ongoing removal of a maximum number of cats for adoption, and the allowance of time for natural attrition to occur. Successful TNR programmes have been reported in a limited number of studies where the programme has been targeted and managed closely. For example Levy, et al. [63] reported that over an 11 year period a colony population was successfully reduced by 66%, but this successfulness included the removal and adoption of 47% of the original population and the continual de-sexing of new arrivals to the colony before breeding could occur. In a further study by Stoskopf and Nutter [64], six de-sexed colonies showed a mean decrease in population of 36% during the first two years of study. Conversely, other examples of TNR reported in the scientific literature suggest that TNR is an ineffective mechanism for the population control of stray cats, in particular those that are unmanaged. For example Foley*,* et al. [65] reported that TNR was not successful at reducing the population numbers of stray cats in San Diego County, California and Alachua County, Florida and concluded that a reduction in growth per capita did not occur because the critical value of a 71–94% de-sexing rate was not achieved. Other research has used modeling to come up with similar figures of 75% or greater proportions of stray cats requiring de-sexing for population control to be effective [66]. In Rome, over the course of a decade, a 32% decrease in the population size over 103 colonies was observed, but the effectiveness of the TNR effort was substantially reduced by a 21% immigration rate of cats into these colonies from the companion cat population [67]. Furthermore, the successes of TNR programmes based on the impact on wildlife have not be reported. The successfulness of TNR would therefore seem to depend on how the programme is applied and managed, with scientific literature suggesting that TNR would likely be unsuccessful for cats categorised as unmanaged or feral in the present study. Consequently, we may have observed differing levels of support for TNR in the present study if it had been presented as a management strategy that requires intensive management and effort to remove and adopt cats as part of the TNR programme, and our results relating to TNR should be interpreted with caution.

### 4.4. Limitations

Aside from the limitations previously mentioned, it is important to point out that the majority of our participants were well educated. The proportion of participants with tertiary education was much higher than that represented in the most recent New Zealand census, potentially biasing our results. Additionally, although we gained participation from individuals living in locations in similar proportions to the recent New Zealand census (e.g., urban, suburban, and rural), the locations in which we collected responses were restricted to cities and towns in the upper North Island. Furthermore, although we utilised a standardised sampling method [36], we had no control over participants who independently chose to approach and participate. Consequently, the representative nature of our findings to the New Zealand population as a whole must be considered with caution. On the other hand, as discussed, our findings often parallel public opinion expressed regarding cat management both nationally and internationally, and therefore it is likely that similar trends would be observed in the lower North Island and throughout the South Island of New Zealand. Further research in these geographical areas is needed to confirm this.

## 5. Conclusions

To conclude, the results of this study indicate that the New Zealand public is largely in favour of a National Cat Management Strategy to control both owned and unowned cats and to aid in the reduction of cat predation, with high levels of concern expressed regarding the predation of both non-native and native wildlife. The findings of this research provide useful insights into public acceptance of a range of management strategies and the demographic variables that influence these. In particular, older participants expressed increased concern regarding the predation of wildlife by cats and were generally more in favour of the management techniques for owned cats and lethal methods of control for unowned cats. This suggests that older individuals may be more willing to comply with a National Cat Management Strategy, while younger indivdiuals may benefit from educational drives that detail the benefits for both wildlife populations and cat populations.

The differences observed between males and females suggests that females are more concerned about the humane treatment of individual animals as opposed to overall species conservation. Consequently, to increase female buy-in, the management techniques posited within a National Strategy must be demonstrated to be humane and considerate of individual animal welfare. Finally, the benefits of a National Cat Management Strategy must illuminate the differing benefits of cat manangement for both owners and non owners.

## Figures and Tables

**Table 1:** Participant demographics (*n* = 1011) showing a comparison of survey sample sizes with New Zealand national statistics.

Demographic	Demographic Categories	N	Survey Sample %	New Zealand Census 2013%
Age	18−25 years	253	25%	14%
	26−35 year	217	21%	16%
	36−45 years	132	13%	18%
	46−55 years	134	13%	19%
	56−65 years	135	13%	15%
	65+ years	138	14%	18%
Gender	Male	497	49%	49%
	Female	494	49%	51%
Ethnicity	New Zealand European	515	51%	64%
	Māori	70	7%	15%
	Asian/Indian	124	12%	12%
	European	188	19%	8%
	Pacific Cook Island	25	2%	7%
	Other	79	8%	2%
Marital Status	Single	457	45%	35%
	Married	327	32%	48%
	Divorced	52	5%	11%
	De facto	140	14%	-
	Widowed	33	3%	6%
Residential Location	Urban	332	32%	72% ^#^
	Suburban	427	42%	-
	Rural	145	14%	14% ^#^
Income	<$50,000 per annum	277	27%	6%
	$50,000–$100,000 per annum	262	26%	21%
	>$100,000	117	12%	6%
	No answer	52	5%	10%
Education	No formal education/Primary	19	2%	21%
	Secondary	224	22%	33%
	Certificate/Diploma	230	23%	29%
	Undergraduate	296	29%	14%
	Postgraduate	229	23%	6%
Employed	Yes	708	70%	61%
	No	301	30%	39%
Cat Owner *	Yes	326	32%	44%
	No	684	68%	56%

Total *n* for each demographic differs from the total survey population as a result of non-response from participants. %’s are calculated based on the total number of respondents (*n* = 1011). Source: [37]; * Source: [1]; ^#^ Based on 2006 census data.

## Supplementary Materials

The following are available online at www.mdpi.com/2076-2615/7/7/49/s1, Table S1: The influence of demographic variables on responses to questions regarding cat predation, Table S2: The influence of demographic variables on responses to questions surrounding responsible companion cat ownership, Table S3: The influence of demographic variables on responses to questions regarding the management of cat populations.
