# Supplementary material for: A Survey of Public Opinion on Cat (Felis catus) Predation and the Future Direction of Cat Management in New Zealand
Source: Animals (Basel). 2017 Jul 3;7(7):49. doi: 10.3390/ani7070049 (PMC5532564; doi:10.3390/ani7070049)
Supplement: Supplementary file 1 [file animals-07-00049-s001.pdf]

Table S1: The influence of demographic variables on responses to questions regarding cat predation

				Question Response				
Question: Are you concerned about the predation of	Model Diagnostics	Demographic		Yes	No	Don't Know	Coefficient (Z)	Odds Ratio(CI)
Non-native wildlife by companion cats?	1. Log-Likelihood *p* Value = 0.02	Ethnicity	NZ European	179 (35%)	322 (62%)	14 (3%)		
			NZ Maori	27 (39%)	38 (54%)	5 (7%)		
			Asian/Indian	63 (51%)	52 (42%)	9 (7%)	-4.04	0.28 (0.15-0.52)
	2. Pearson Chi -Squared = 0.56		European	72 (38%)	107 (57%)	9 (5%)		
			Pacific/Cook Is.	10 (40%)	13 (52%)	2 (8%)		
	3. Deviance Chi-squared = 1.00		Other	30 (38%)	44 (56%)	5 (6%)		
		Age (median)		46-55years	26-35years	26-35years	-4.34	0.71 (0.61-0.83)
Non-native wildlife by colony cats?	1. Log-Likelihood *p* Value = <0.0001	Ethnicity	NZ European	270 (52%)	230 (45%)	15 (3%)		
			NZ Maori	36 (52%)	31 (44%)	3 (4%)		
			Asian/Indian	79 (65%)	36 (29%)	7 (6%)	-3.29	0.34 (0.18-0.65)
	2. Pearson Chi -Squared = 1.00		European	98 (52%)	84 (45%)	6 (3%)		
			Pacific/Cook Is.	11 (44%)	12 (48%)	2 (8%)		
			Other	45 (57%)	30 (38%)	4 (5%)		
	3. Deviance Chi-squared = 1.00	Age (median)		36-45years	26-35years	26-35years	-3.55	0.76 (0.65-0.88)
		Marital Status	Single	236 (52%)	200 (44%)	21 (4%)		
			Married	185 (57%)	130 (40%)	12 (4%)	2.91	8.29 (2.00-34.40)
			Divorced	39 (75%)	12 (23%)	1 (2%)		
			De facto	63 (45%)	73 (52%)	4 (3%)		
			Widowed	22 (67%)	9 (27%)	2 (6%)		
		Income	Less than $50,000	132 (48%)	134 (48%)	11 (4%)		
			$50,000-$100,000	143 (54%)	112 (43%)	7 (3%)		
			$100,000+	67 (57%)	48 (41%)	2 (2%)		
			No answer	24 (46%)	24 (46%)	4 (8%)	2.33	7.27 (1.37-38.62)
Non-native wildlife by unmanaged stray cats?	1. Log-Likelihood *p* Value = 0.014	Age (median)		36-45years	26-35years	26-35years	-3.28	0.77 (0.66-0.90)
		Ethnicity	NZ European	292 (57%)	217 (42%)	6 (1%)		
			NZ Maori	41 (59%)	27 (38%)	2 (3%)		
	2. Pearson Chi -Squared = 1.00		Asian/Indian	87 (70%)	32 (26%)	5 (4%)	-2.85	0.39 (0.21-0.75)
			European	106 (69%)	40 (26%)	7 (5%)		
			Pacific/Cook Is.	18 (72%)	6 (24%)	1 (4%)		
	3. Deviance Chi-squared = 1.00		Other	49 (62%)	27 (34%)	3 (4%)		
		Marital Status	Single	261 (57%)	186 (41%)	10 (2%)		
			Married	199 (61%)	120 (37%)	8 (2%)	1.96	5.58 (1.00-34.22)
			Divorced	40 (77%)	9 (17%)	3 (6%)		
			De facto	73 (52%)	64 (46%)	3 (2%)		
			Widowed	26 (79%)	6 (18%)	1 (3%)		
		Residential Location	Urban	196 (59%)	128 (39%)	8 (2%)		
			Suburban	251 (59%)	165 (39%)	11 (2%)		
			Rural	89 (61%)	52 (36%)	4 (3%)	1.98	6.64 (1.02-43.21)
Non-native wildlife by feral cats?	1. Log-Likelihood *p* Value = 0.006	Age (median		36-45years	26-35years	36-45years	-3.10	0.78 (0.67-0.91)
		Ethnicity	NZ European	303 (59%)	192 (37%)	20 (4%)		
			NZ Maori	46 (66%)	22 (31%)	2 (3%)		
	2. Pearson Chi -Squared = 0.73		Asian/Indian	74 (60%)	42 (34%)	8 (6%)	-2.56	0.43 (0.23-0.82)
			European	118 (63%)	65 (35%)	5 (2%)		
			Pacific/Cook Is.	17 (68%)	7 (28%)	1 (4%)		
	3. Deviance Chi-squared = 1.00		Other	43 (54%)	30 (38%)	6 (8%)		
		Education	Primary (or less)	12 (63%)	5 (26%)	2 (11%)		

			Secondary	133 (59%)	85 (38%)	6 (3%)	-2.0	0.05 (0.00-0.95)
			Certificate/Diploma	145 (63%)	75 (33%)	10 (4%)		
			Undergraduate	168 (57%)	112 (38%)	16 (5%)		
			Postgraduate	143 (63%)	78 (34%)	8 (3%)	-2.07	0.05 (0.00-0.85)
Native wildlife by companion cats?	1. Log-Likelihood *p* Value = 0.003<br>2. Pearson Chi -Squared = 1.00<br>3. Deviance Chi-squared = 1.00	Gender	Male	357 (72%)	132 (26%)	8 (2%)		
			Female	327 (66%)	150 (30%)	17 (4%)	2.07	3.81 (1.08 -3.49)
		Age (median)		36-45years	26-35years	36-45years	-4.39	0.67 (0.55-0.80)
Native wildlife by colony cats?	1. Log-Likelihood *p* Value = 0.003<br>2. Pearson Chi -Squared = 1.00<br>3. Deviance Chi-squared = 1.00	Ethnicity	NZ European	437 (85%)	66 (13%)	12 (2%)		
			NZ Maori	55 (79%)	14 (20%)	1 (1%)		
			Asian/Indian	95 (77%)	23 (18%)	6 (5%)		
			European	147 (78%)	34 (18%)	7 (4%)		
			Pacific/Cook Is.	21 (84%)	4 (16%)	0 (0%)		
			Other	61 (77%)	17 (22%)	1 (1%)	2.16	2.55 (1.09-5.94)
		Age		36-45years	26-35years	26-35years	-2.89	0.71 (0.57-0.90)
		Education	Primary (or less)	12 (63%)	6 (32%)	1 (5%)		
			Secondary	174 (78%)	44 (19%)	6 (3%)		
			Certificate/Diploma	192 (84%)	33 (14%)	5 (2%)	-2.07	0.14 (0.02-0.90)
			Undergraduate	237 (80%)	45 (15%)	14 (5%)	-2.32	0.11 (0.02-0.71)
			Postgraduate	200 (87%)	27 (12%)	2 (1%)	-2.40	0.10 (0.01-0.65)
		Income	Less than $50,000	216 (78%)	54 (19%)	7 (3%)		
			$50,000-$100,000	227 (87%)	31 (12%)	4 (1%)		
			$100,000+	96 (82%)	19 (16%)	2 (2%)		
			No answer	43 (83%)	5 (9%)	4 (8%)	2.45	9.24 (1.56-54.57)
Native wildlife by unmanaged stray cats?	1. Log-Likelihood *p* Value = 0.001<br>2. Pearson Chi -Squared = 1.00<br>3. Deviance Chi-squared = 1.00	Age (median		36-45years	26-35years	26-35years	-3.41	0.61 (0.46-0.81)
		Income	Less than $50,000	233 (84%)	37 (13%)	7 (3%)		
			$50,000-$100,000	239 (91%)	23 (9%)	0 (0%)		
			$100,000+	101 (86%)	14 (12%)	2 (2%)	2.40	3.09 (1.23-7.73)
			No answer	46 (88%)	3 (6%)	3 (6%)		

Table S2: The influence of demographic variables on responses to questions surrounding responsible companion cat ownership

Question		Demographic		Question Response: Yes	No	Don't Know	Coefficient (Z)	Odds Ratio(CI)
Should it be compulsory for companion cats to be desexed?	1. Log-Likelihood *p* Value = < 0.0001<br>2. Pearson Chi-Squared = 0.93<br>3. Deviance Chi-squared = 1.00	Gender	Male	248 (50%)	212 (43%)	37 (7%)		
			Female	323 (65%)	144 (29%)	27 (6%)	-2.16	0.66 (0.45-1.96)
		Age (median)		46-55years	26-35years	36-45years	-4.47	0.68 (0.58-0.81)
Should it be compulsory for companion cats to be microchipped?	1. Log-Likelihood *p* Value = 0.05<br>2. Pearson Chi-Squared = 0.92<br>3. Deviance Chi-squared = 1.00	Residential Location	Urban	223 (67%)	91 (27%)	18 (6%)	2.37	2.95 (1.21-7.20)
			Suburban	279 (65%)	113 (27%)	35 (8%)		
			Rural	94 (65%)	48 (33%)	3 (2%)		
		Age (median)		36-45years	26-35years	46-55years	2.35	1.43 (1.06-1.92)
		Marital Status	Single	284 (62%)	145 (32%)	28 (6%)		
			Married	225 (69%)	83 (25%)	19 (6%)	-2.25	0.54 (0.32-0.92)
			Divorced	37 (71%)	14 (27%)	1 (2%)		
			De facto	95 (68%)	37 (26%)	8 (6%)		
			Widowed	25 (76%)	5 (15%)	3 (9%)		
Should it be compulsory for companion cats to be registered with the council?	1. Log-Likelihood *p* Value = <0.0001<br>2. Pearson Chi-Squared = 0.05<br>3. Deviance Chi-squared =1.00	Ethnicity	NZ European	282 (55%)	208 (40%)	25 (5%)		
			NZ Maori	38 (54%)	28 (40%)	4 (6%)		
			Asian/Indian	108 (87%)	14 (11%)	2 (2%)	-3.14	0.18 (0.06-0.52)
			European	106 (57%)	72 (38%)	10 (5%)		
			Pacific/Cook Is.	16 (70%)	6 (26%)	1 (4%)		
			Other	59 (75%)	19 (24%)	1 (1%)		
		Cat Owner	Yes	153 (47%)	162 (50%)	11 (3%)		
			No	463 (68%)	186 (27%)	35 (5%)	-3.89	0.46 (0.31-0.68)
		Age (median)		36-45years	46-55years	26-35years	4.53	1.45 (1.24-1.71)
Should there be a limit to the number of cats one household can own at a time?	1. Log-Likelihood *p* Value = 0.002<br>2. Pearson Chi-Squared = 0.09<br>3. Deviance Chi-squared = 1.00	Ethnicity	NZ European	392 (76%)	99 (19%)	24 (5%)		
			NZ Maori	46 (66%)	21 (30%)	3 (4%)		
			Asian/Indian	73 (59%)	47 (38%)	4 (3%)		
			European	124 (66%)	54 (29%)	9 (5%)	2.22	1.79 (1.07-3.00)
			Pacific/Cook Is.	13 (52%)	11 (44%)	1 (4%)		
			Other	52 (66%)	24 (30%)	3 (4%)		
		Age (median)		36-45years	26-35years	26-35years	-1.99	0.83 (0.69-1.00)
		Marital Status	Single	267 (58%)	168 (37%)	21 (5%)		
			Married	261 (80%)	52 (16%)	14 (4%)	-2.61	0.47 (0.27-0.83)
			Divorced	42 (81%)	8 (15%)	2 (4%)		
			De facto	102 (73%)	31 (22%)	7 (5%)		
			Widowed	32 (97%)	1 (3%))	0 (0%)		
		Education	Primary (or less)	12 (63%)	5 (26%)	2 (11%)		
			Secondary	150 (67%)	62 (28%)	12 (5%)		
			Certificate/Diploma	166 (72%)	56 (24%)	8 (4%)	-2.17	0.06 (0.00-0.76)
			Undergraduate	200 (68%)	83 (28%)	13 (4%)		
			Postgraduate	167 (73%)	52 (23%)	9 (4%)		
Should there be times when cats must be confined to the owners	1. Log-Likelihood *p* Value =	Cat Owner	Yes	101 (31%)	202 (62%)	23 (7%)		
			No	316 (46%)	308 (45%)	59 (9%)	-2.64	0.59 (0.40-0.87)
		Ethnicity	NZ European	181 (35%)	291 (57%)	42 (8%)		

property	<0.0001		NZ Maori	31 (44%)	36 (52%)	3 (4%)	-2.06	0.48 (0.24-0.96)
			Asian/Indian	70 (56%)	38 (31%)	16 (13%)	-4.82	0.20 (0.10-0.38)
	2. Pearson Chi -Squared = 0.13		European	76 (59%)	38 (29%)	16 (12%)		
			Pacific/Cook Is.	16 (64%)	8 (32%)	1 (4%)	-2.20	0.26 (0.08-0.86)
	3. Deviance Chi-squared = 0.99		Other	37 (47%)	38 (48%)	4 (5%)		
Should there be times when cats must be confined inside their owner's home?	1. Log-Likelihood p Value = 0.001	Ethnicity	NZ European	171 (33%)	319 (62%)	24 (5%)		
			NZ Maori	26 (37%)	42 (60%)	2 (3%)		
			Asian/Indian	61 (49%)	56 (45%)	7 (6%)	-4.06	0.27 (0.14-0.51)
			European	63 (34%)	107 (57%)	17 (9%)		
	2. Pearson Chi -Squared = 0.23		Pacific/Cook Is.	14 (56%)	10 (40%)	1 (4%)	-2.90	0.17 (0.05-0.56)
			Other	28 (35%)	42 (53%)	9 (11%)		
		Age (median)		36-45years	26-35years	36-45years	-4.20	0.71 (0.60-0.83)
	3. Deviance Chi-squared = 1.00	Income	Less than $50,000	90 (32%)	169 (61%)	18 (7%)		
			$50,000-$100,000	88 (34%)	156 (59%)	18 (7%)		
			$100,000+	29 (25%)	83 (71%)	5 (4%)	2.35	2.09 (1.13-3.87)
			No answer	21 (40%)	27 (52%)	1 (8%)		
Should there be areas in which companion cats are not allowed to be owned?	1. Log-Likelihood p Value = < 0.0001	Cat Owner	Yes	189 (58%)	109 (33%)	28 (9%)		
			No	482 (71%)	165 (24%)	37 (5%)	-4.68	0.37 (0.24-0.56)
		Ethnicity	NZ European	350 (68%)	130 (25%)	35 (7%)		
			NZ Maori	44 (63%)	19 (27%)	7 (10%)		
			Asian/Indian	80 (64%)	37 (30%)	7 (6%)	2.31	2.22 (1.13-4.36)
	2. Pearson Chi -Squared = 0.17		European	122 (65%)	55 (29%)	11 (6%)	2.09	1.17 (1.03-2.82)
			Pacific/Cook Is.	15 (60%)	8 (32%)	2 (8%)		
			Other	51 (64%)	25 (32%)	3 (4%)		
	3. Deviance Chi-squared = 1.00	Income	Less than $50,000	185 (67%)	63 (23%)	29 (10%)		
			$50,000-$100,000	188 (72%)	63 (24%)	11 (4%)	-2.66	0.33 (0.15-0.75)
			$100,000+	76 (65%)	36 (31%)	5 (4%)	-2.14	0.27 (0.08-0.90)
			No answer	29 (56%)	17 (33%)	6 (11%)		

Table S3: The influence of demographic variables on responses to questions regarding the management of cat populations

Question:	Demographic		Question Response					Coefficient (Z)	Odds Ratio(CI)
Who should be			Government	Council[2]	SPCA[3]	Combination[4]	Other		
responsible for	Cat Owner	Yes	31 (12%)	91 (35%)	24 (9%)	85 (33%)	29 (11%)		
controlling colony cats?		No	73 (13%)	187 (33%)	63 (11%)	188 (33%)	53 (10%)	1.98	2.54 (1.01-6.37)
	Ethnicity	NZ European	46 (11%)	171 (39%)	40 (9%)	133 (31%)	45 (10%)		
Model Diagnostics		NZ Maori	7 (12.5%)	14 (25%)	8 (14%)	20 (36%)	7 (12.5%)		
1. Log-Likelihood		Asian/Indian	18 (18%)	29 (29%)	12 (12%)	34 (34%)	6 (6%)	-2.29	0.27 (0.09-0.83)
p Value = 0.004		European	20 (13%)	52 (35%)	16 (11%)	48 (32%)	14 (9%)	-2.14	0.40 (0.18-0.93)
		Pacific/Cook Is.	3 (17%)	2 (11%)	2 (11%)	9 (50%)	2 (11%)		
2. Pearson Chi		Other	10 (17%)	9 (15%)	7 (12%)	25 (42%)	8 (14%)	-2.32	0.06 (0.01-0.65)
-Squared = 0.64	Residential	Urban	43 (16%)	79 (30%)	25 (10%)	87 (33%)	28 (11%)		
	Location	Suburban	33 (9%)	124 (35%)	42 (12%)	125 (35%)	30 (9%)	[2]2.06	2.13 (1.04-4.36)
3. Deviance Chi-squared								[3]2.52	3.57 (1.33-9.57)
= 1.00								[4]2.50	2.43 (1.21-4.89)
		Rural	13 (11%)	38 (32%)	11 (9%)	40 (33%)	18 (15%)		
	Income	Less than $50,000	26 (12%)	59 (27%)	32 (15%)	84 (38%)	17 (8%)		
		$50,000-$100,000	21 (9%)	80 (36%)	16 (7%)	87 (39%)	21 (9%)	-2.14	0.32 (0.11-0.89)
		$100,000+	13 (13%)	36 (37%)	6 (6%)	30 (31%)	13 (13%)	-2.29	0.19 (0.05-0.79)
		No answer	7 (16%)	14 (33%)	2 (5%)	16 (37%)	4 (9%)		
What action should be taken towards			Lethal Action	TNR[2]	Non Killing Method[3]	Other	Don't Know		
controlling colony cats?	Cat Owner	Yes	54 (20%)	101 (38%)	55 (21%)	51 (19%)	4 (2%)	[3]-2.24	0.46 (0.23-0.91)
								[2]-2.15	0.53 (0.29-0.95)
Model Diagnostics		No	140 (25%)	214 (37%)	82 (14%)	112 (20%)	24 (4%)		
1. Log-Likelihood	Gender	Male	101 (25%)	139 (35%)	58 (15%)	90 (22%)	13 (3%)		
p Value = <0.0001		Female	90 (22%)	172 (41%)	74 (18%)	68 (16%)	14 (3%)	2.22	2.11 (1.09-4.09)
	Age (median)		56-65years	26-35years	36-45years	26-35years	26-35years	-3.72	0.61 (0.47-0.79)
2. Pearson Chi	Income	Less than $50,000	26 (12%)	104 (48%)	35 (16%)	49 (22%)	5 (2%)		
-Squared = 0.13		$50,000-$100,000	60 (26%)	80 (35%)	34 (15%)	49 (21%)	6 (3%)	-2.73	0.39 (0.20-0.77)
		$100,000+	34 (35%)	24 (25%)	20 (21%)	18 (18%)	1 (1%)	-2.73	0.30 (0.12-0.71)
3. Deviance Chi-squared		No answer	12 (27%)	17 (39%)	9 (21%)	5 (11%)	1 (2%)		
= 1.00									
	Residential	Urban	57 (21%)	102 (38%)	39 (15%)	59 (22%)	11 (4%)		
	Location	Suburban	71 (20%)	147 (41%)	64 (18%)	64 (18%)	10 (3%)		
		Rural	46 (38%)	35 (29%)	20 (16.5%)	20 (16.5%)	0 (0%	-3.20	0.27 (0.12-0.61)
Should action be taken			Yes		No		Don't Know		
towards controlling	Age (median)		36-45years		26-35years		26-35years	-2.46	0.64 (0.45-0.91)
unmanaged stray cats?	Ethnicity	NZ European	487 (93%)		24 (5%)		13 (2%)		
		NZ Maori	63 (90%)		3 (4%)		4 (6%)		
Model Diagnostics		Asian/Indian	104 (84%)		13 (10%)		7 (6%)		
1. Log-Likelihood		European	165 (88%)		18 (9%)		5 (3%)		
p Value =		Pacific/Cook Is.	21 (84%)		2 (8%)		2 (8%)		
		Other	65 (82%)		13 (17%)		1 (1%)	2.85	4.88 (1.64-14.54)
2. Pearson Chi	Income	Less than $50,000	248 (90%)		17 (6%)		12 (4%)		
-Squared =		$50,000-$100,000	243 (93%)		15 (6%)		4 (1%)		
		$100,000+	103 (88%)		13 (11%)		1 (1%)	3.21	6.80 (2.11-21.96)
3. Deviance Chi-squared =		No answer	49 (94%)		1 (2%)		2 (4%)		
Who should be			Government	Council	SPCA	Combination	Other		
responsible for	Gender	Male	57 (13%)	134 (32%)	46 (11%)	136 (32%)	50 (12%)		
controlling unmanaged		Female	62 (14%)	130 (29%)	48 (11%)	165 (37%)	39 (9%)	-2.34	0.38 (0.17-0.85)
stray cats?	Ethnicity	NZ European	54 (12%)	161 (34%)	43 (9%)	159 (34%)	53 (11%)		
		NZ Maori	8 (13%)	15 (25%)	13 (22%)	17 (28%)	7 (12%)		
Model Diagnostics		Asian/Indian	23 (23%)	26 (26%)	12 (12%)	33 (32%)	7 (7%)	-2.65	0.14 (0.02-0.78)

1. Log-Likelihood		European	23 (14%)	54 (34%)	17 (11%)	53 (33%)	13 (8%)		
p Value = 0.004		Pacific/Cook Is.	2 (9.5%)	5 (24%)	2 (9.5%)	11 (52%)	1 (5%)		
		Other	10 (15%)	7 (11%)	9 (14%)	31 (48%)	8 (12%)		
2. Pearson Chi	Residential	Urban	50 (18%)	79 (28%)	31 (11%)	92 (32%)	32 (11%)		
-Squared = 1.00	Location	Suburban	41 (11%)	120 (32%)	46 (12%)	141 (37%)	31 (8%)	2.29	2.12 (1.11-4.04)
		Rural	16 (13%)	35 (27%)	13 (10%)	44 (34%)	20 (16%)		
3. Deviance Chi-squared	Income	Less than $50,000	30 (12%)	60 (25%)	34 (14%)	101 (41%)	13 (8%)		
= 1.00		$50,000-$100,000	24 (10%)	73 (31%)	24 (10%)	92 (39%)	25 (10%)		
		$100,000+	15 (15%)	39 (38%)	5 (5%)	29 (28%)	14 (14%)	-2.68	0.14 (0.03-0.59)
		No answer	10 (21%)	14 (29%)	5 (10%)	17 (36%)	2 (4%)		
What action should be taken towards controlling unmanaged stray cats?			Lethal Action	TNR[2]	Non Killing Method[3]	Other[4]	Don't Know[5]		
	Gender	Male	130 (30%)	134 (31%)	50 (12%)	101 (23%)	17 (4%)		
		Female	129 (28%)	131 (29%)	84 (19%)	90 (20%)	20 (4%)	3.88	3.60 (1.88-6.87)
	Age (median)							[2]-5.55	0.54 (0.44-0.67)
Model Diagnostics								[3]-4.60	0.53 (0.41-0.70)
1. Log-Likelihood			56-65years	26-35years	26-35years	36-45years	26-35years	[4]-4.09	0.62 (0.49-0.78)
p Value = < 0.0001								[5]-3.22	0.39 (0.22-0.69)
	Ethnicity	NZ European	178 (37%)	135 (28%)	65 (14%)	86 (18%)	14 (3%)		
2. Pearson Chi		NZ Maori	15 (24%)	26 (41%)	9 (14%)	9 (14%)	4 (7%)		
-Squared = 0.68		Asian/Indian	9 (9%)	39 (37%)	17 (16%)	33 (32%)	6 (6%)	[3]1.97	4.17 (1.01-17.26)
								[4]2.40	5.14 (1.35-19.60)
3. Deviance Chi-squared		European	45 (27%)	45 (27%)	30 (18%)	38 (23%)	7 (4%)	2.01	2.17 (1.02-4.62)
= 1.00		Pacific/Cook Is.	2 (10%)	3 (14%)	4 (19%)	9 (43%)	3 (14%)		
		Other	14 (22%)	17 (26%)	13 (20%)	17 (26%)	4 (6%)		
	Marital Status	Single	62 (16%)	132 (34%)	77 (20%)	89 (23%)	27 (7%)		
		Married	121 (39%)	82 (27%)	36 (12%)	64 (21%)	5 (5%)		
		Divorced	23 (49%)	10 (21%)	2 (5%)	11 (23%)	1 (2%)		
		De facto	42 (33%)	39 (30%)	20 (16%)	23 (18%)	4 (3%)	[2]-2.72	0.37 (0.18-0.76)
								[3]-3.08	0.25 (0.10-0.61)
		Widowed	15 (46%)	5 (15%)	4 (12%)	8 (24%)	1 (3%)		
	Residential	Urban	75 (26%)	82 (29%)	44 (15%)	67 (23%)	20 (7%)		
	Location	Suburban	105 (27%)	118 (30%)	63 (16%)	89 (23%)	15 (4%)		
		Rural	61 (47%)	29 (23%)	16 (12%)	22 (17%)	1 (1%)	-1.96	0.44 (0.20-1.00)
	Income	Less than $50,000	39 (14%)	99 (36%)	47 (17%)	53 (19%)	39 (14%)		
		$50,000-$100,000	73 (30%)	74 (30%)	32 (13%)	55 (23%)	9 (4%)	-2.04	0.06 (0.00-0.90)
		$100,000+	52 (50%)	19 (18%)	13 (13%)	18 (18%)	1 (1%)	-3.13	0.28 (0.12-0.62)
		No answer	14 (29%)	12 (25%)	11 (22%)	8 (16%)	4 (8%)		
Should action be taken towards controlling feral cats?				Yes	No		Don't Know		
	Age (median)			36-45years	18-25years		26-35years	-4.01	0.46 (0.32-0.67)
	Ethnicity	NZ European		461 (89%)	29 (6%)		25 (5%)		
Model Diagnostics		NZ Maori		61 (87%)	6 (9%)		3 (4%)		
1. Log-Likelihood		Asian/Indian		87 (70%)	28 (23%)		9 (7%)	2.01	2.73 (1.03-7.26)
p Value = 0.004		European		162 (86%)	16 (9%)		10 (5%)		
		Pacific/Cook Is.		17 (68%)	5 (20%)		3 (12%)		
2. Pearson Chi		Other		62 (78%)	15 (19%)		2 (3%)	2.54	3.71 (1.35-10.22)
-Squared = 0.22									
3. Deviance Chi-squared = 1.00									
Who should be responsible for controlling feral cats?			Government	Council	SPCA	Combination	Other		
	Ethnicity	NZ European	97 (21%)	114 (25%)	25 (6%)	142 (31%)	77 (17%)		
		NZ Maori	12 (22%)	13 (23%)	5 (9%)	18 (32%)	8 (14%)		
		Asian/Indian	24 (28%)	18 (21%)	12 (14%)	26 (31%)	5 (6%)	-2.13	0.17 (0.03-1.10)

Model Diagnostics		European	36 (23%)	39 (24%)	13 (8%)	52 (33%)	20 (18%)		
1. Log-Likelihood		Pacific/Cook Is.	5 (29%)	2 (12%)	0 (0%)	10 (59%)	0 (0%)		
p Value = 0.06		Other							
2. Pearson Chi -Squared = 0.36									
3. Deviance Chi-squared = 1.00									
What action should be taken towards controlling feral cats?			Lethal Action	TNR[2]	Non Killing Method[3]	Other[4]	Don't Know+5		
	Gender	Male	165 (40%)	105 (25%)	23 (6%)	98 (24%)	21 (5%)		
		Female	159 (37%)	117 (28%)	31 (7%)	95 (22%)	24 (6%)	2.09	1.72 (1.04-2.86)
Model Diagnostics	Age (median)		46-55years	26-35years	26-35years	36-45years	26-35years	-4.92	0.57 (0.46-0.72)
1. Log-Likelihood	Ethnicity	NZ European	232 (50%)	106 (23%)	21 (5%)	85 (19%)	16 (3%)		
p Value = < 0.0001		NZ Maori	16 (26%)	20 (33%)	4 (7%)	18 (29%)	3 (5%)	2.33	2.98 (1.19-7.50)
		Asian/Indian	11 (13%)	23 (26%)	6 (7%)	34 (39%)	13 (15%)	2.73	4.16 (1.49-11.59)
2. Pearson Chi								2.33	5.34 (1.30-21.90)
-Squared = 0.92		European	62 (39%)	41 (25%)	18 (11%)	32 (20%)	8 (5%)	3.41	4.98 (1.98-12.53)
		Pacific/Cook Is.	2 (12%)	3 (17.5%)	3 (17.5%)	7 (41%)	2 (12%)	2.19	12.49 (1.30-
3. Deviance Chi-squared								2.42	119.61)
= 1.00									27 (1.87-389.09)
		Other	7 (12%)	30 (50%)	2 (3%)	18 (30%)	3 (5%)	3.00	6.91 (1.96-24.37)
	Marital Status	Single	88 (24%)	122 (34%)	31 (9%)	90 (25%)	30 (8%)		
		Married	155 (53%)	65 (22%)	9 (3%)	57 (19%)	8 (3%)	-3.01	0.16 (0.05-0.53)
		Divorced	26 (55%)	5 (11%)	3 (6%)	13 (28%)	0 (0%)		
		De facto	49 (42%)	26 (22.5%)	10 (9%)	26 (22.5%)	5 (4%)	-2.46	0.40 (0.19-0.83)
		Widowed	14 (43%)	8 (24%)	0 (0%)	9 (27%)	2 (6%)		
